# Modelling Pancreatic Neuroendocrine Cancer: From Bench Side to Clinic

**DOI:** 10.3390/cancers12113170

**Published:** 2020-10-28

**Authors:** Alexander Ney, Gabriele Canciani, J. Justin Hsuan, Stephen P. Pereira

**Affiliations:** 1 Institute for Liver and Digestive Health, University College London, London NW3 2PF, UK; Alexander.ney.15@ucl.ac.uk (A.N.); canciani.1679741@studenti.uniroma1.it (G.C.); j.hsuan@ucl.ac.uk (J.J.H.); 2 School of Medicine, La Sapienza University, 00185 Rome, Italy

**Keywords:** pancreatic cancer, pancreatic neuroendocrine tumours, disease models, genetically engineered mouse models, multicellular spheroids, organoids

## Abstract

**Simple Summary:**

Although rare, pancreatic neuroendocrine tumours (pNETs) represent the second most common group of pancreatic neoplasms, and associated patient outcomes remain largely poor. Advances in diagnosis and management rely strongly on the ability to accurately model these tumours ex vivo; however, currently available models are limited. Moreover, the importance of the extracellular matrix in disease development and progression should be heavily considered when modelling the disease. This review will outline the most clinically relevant disease models of pNETs and challenges in their use, as well as recent advances and future directions in their modelling.

**Abstract:**

Pancreatic neuroendocrine tumours (pNETs) are a heterogeneous group of epithelial tumours with neuroendocrine differentiation. Although rare (incidence of <1 in 100,000), they are the second most common group of pancreatic neoplasms after pancreatic ductal adenocarcinoma (PDAC). pNET incidence is however on the rise and patient outcomes, although variable, have been linked with 5-year survival rates as low as 40%. Improvement of diagnostic and treatment modalities strongly relies on disease models that reconstruct the disease ex vivo. A key constraint in pNET research, however, is the absence of human pNET models that accurately capture the original tumour phenotype. In attempts to more closely mimic the disease in its native environment, three-dimensional culture models as well as in vivo models, such as genetically engineered mouse models (GEMMs), have been developed. Despite adding significant contributions to our understanding of more complex biological processes associated with the development and progression of pNETs, factors such as ethical considerations and low rates of clinical translatability limit their use. Furthermore, a role for the site-specific extracellular matrix (ECM) in disease development and progression has become clear. Advances in tissue engineering have enabled the use of tissue constructs that are designed to establish disease ex vivo within a close to native ECM that can recapitulate tumour-associated tissue remodelling. Yet, such advanced models for studying pNETs remain underdeveloped. This review summarises the most clinically relevant disease models of pNETs currently used, as well as future directions for improved modelling of the disease.

## 1. Introduction

Pancreatic neuroendocrine tumours (pNETs) are the second most common subtype of primary pancreatic tumours and represent around 1–2% of pancreatic neoplasms despite their relative rarity (annual incidence approx. 1 in 100,000) [1]. Data obtained from autopsy studies however, suggested that many of these tumours go unnoticed due to their indolent course, and their incidence could be as high as 10% [2]. pNETs are epithelial neoplasms that arise from the endocrine portion of the pancreas (islets of Langerhans) and are therefore diverse in both their histological characteristics and their clinical manifestations. Most pNETs arise sporadically; however, some occur as part of inherited (familial) syndromes such as multiple endocrine neoplasia 1 (MEN-1) [3,4]. pNETs can be divided into functioning (fpNETs) and nonfunctioning (NfpNETs) entities, with the latter accounting for nearly 90% of these tumours. NfpNETs produce no clear clinical symptoms until a late stage, while functioning tumours are associated with hormonal syndromes due to their active secretion of biological amines and hormones such as insulin (17%), gastrin (15%), vasoactive intestinal peptide (VIP, 2%), glucagon (1%) and somatostatin (1%) [5,6,7]. Despite recent advances in diagnostics and targeted therapies for these patients, the rarity of this disease and its complex and heterogenous nature, as well as difficulties in accessing patient tumour samples for research purposes, challenge further advancement in improving its detection and overall survival rates [7].

Preclinical disease models offer platforms for studying the complex tumour biology and pathophysiology as well as the effects of different drug treatments in a controlled environment. Popular models include cell lines, patient derived xenograft cells and animal models (i.e., genetically engineered mouse models (GEMMs)), while three-dimensional (3D) models have been a recent focus of attempts to better mimic the in vivo like behaviour of cancer cells and allow the study of complex molecular interactions in cancers (Table S1).

Surrogate models for studying diseases are pivotal yet are not easy to construct in a way that completely recapitulates the complex physiology and cell–cell interactions with both cancerous and noncancerous cells of the tumour microenvironment. Models for studying pNETs are scarce, due in part to the rarity of the disease. However, considering the rise in incidence and the often poor outcomes for patients diagnosed with advanced disease, ongoing development of such models to better mimic and reflect the biology of these tumours are warranted. In this review we summarise the most clinically relevant models of pNETs in current use.

## 2. Pancreatic Neuroendocrine Tumours (pNET) Cell Lines, Orthotopic Models and Primary Cultures

### 2.1. pNET Cell Lines

Available established pNET cell lines include four human, three mouse and two rat cell lines. Animal cell lines have mostly been used for the purpose of studying islet hormone production and their regulation mechanisms in the settings of diabetes mellitus [8], rather than in the context of neuroendocrine cancer, and are therefore beyond the scope of this article.

The most widely used human cell lines are BON-1 and QGP-1. The BON-1 cell line was isolated from a pancreatic serotonin-producing metastatic carcinoid present in a lymph node excised from a 28-year-old male patient [9], while QGP-1 cells were isolated from a primary pancreatic somatostatin-producing pNET in a 61-year-old patient [10].

QGP-1 and BON-1 cells have both been used for the study of molecular processes associated with tumourigenesis (i.e., constitutively activated PI3K/Akt/mTOR signaling pathway) and the effects of their inhibition on disease progression. Both cell lines have been key to studies of current first-line treatments, such as everolimus (RAD001; mTORC1 inhibition) and somatostatin analogues for treating gastroenteropancreatic neuroendocrine neoplasms (GEP-NENs), as well as studies aimed at overcoming drug resistance to these using novel PI3K/mTOR pathway inhibitors [11,12,13,14].

Additional human pNET cell lines include the CM and NT-3 lines. The CM cell line was isolated from the ascitic fluid of a patient with a primary pNET; due to the high resemblance to pancreatic β-cells (in terms of expression of glucose signaling pathways and insulin mRNA expression), it has been used for the study of β-cell function [15]. More recently, the well-differentiated NT-3 cell line was isolated from a lymph node of a male patient with an insulinoma and characterised by Benten et al. [16]. NT-3 cells that were transplanted in recipient mice showed biomolecular features akin to the original tumour such as slow growth and a well-differentiated phenotype, as well as indications of disease progression.

Recent studies aimed at mapping the genomic landscape of BON-1 and QGP-1 cells, however, raise certain issues with respect to their true origin and epithelial/pancreatic-endocrine phenotype.

BON-1 cells bear *P53*, *TSC2* and *NRAS* mutations alongside aberrant activation of the mTOR/Akt pathways, while QGP-1 cells harbour mutations in *P53*, *ATRX* and *SMAD4*, both showing only partial resemblance to commonly occurring pNET genetic mutations [17]. With respect to the BON-1 cell line, it was subsequently suggested that these cells are derived from an adenocarcinoma with features of neuroendocrine differentiation rather than a true pNET [18], and that its use in modelling pNETs is therefore questionable. A more recent analysis published by Hofving et al. is supportive of BON-1 cells indeed having a neuroendocrine origin, with additional mutations potentially arising while in culture [17]. Luley et al. compared the expression of genes associated with epithelial and neuroendocrine differentiation alongside those linked with specific endocrine developmental stages in BON-1, QGP-1 and NT-3 cells [19]. While an epithelial and neuroendocrine phenotype was a feature common to all three, striking differences between BON-1 and QGP-1 compared to NT-3 were found, where the former two expressed genes associated with developmental/progenitor-stage β-cells (i.e., markers such as NEUROD1, NEUROG3, FOXA2). NT-3 cells on the other hand, preferentially expressed markers characteristic of well-differentiated insulinomas (i.e., INS, MAFA) [19]. Lastly, expression patterns of somatostatin receptors 2 and 5 (SSTR 2,5) differed between BON-1, QGP-1 and NT-3 cells, with the latter displaying a 3- and 17-fold increased expression of these receptors compared to BON-1 and QGP-1 cells, respectively [19]. These findings confirm older reports where the expression of SSTR, which is a target for octreotide radionuclide therapy, was found to be relatively low in these cells, suggesting that they are less ideal models for studying the effects of somatostatin analogue treatment compared to NT-3 cells [20,21,22].

### 2.2. Orthotopic Models of pNETs

Two-dimensional (2D) cell culture systems do not take into account the biology and physiology of the original tissue structure. Instead, cells are typically grown in monolayers leading to polarised cell adhesion, as opposed to the three-dimensional (3D) environment of neoplasms. Consequently, gene expression patterns, the complex cell-to-cell interactions and mechanical and biochemical cell signals that occur in vivo are significantly modified [23,24]. Pharmacogenomic differences exist between cells grown in monolayer cultures and those grown on 3D platforms, which in turn influences sensitivity to treatment and hence raise issues around clinical translatability of treatment effects seen in in vivo work [25,26].

Further attempts to create a more realistic tumour environment have led to the popular use of orthotopic transplant models. Implantation of BON-1 and QGP-1 cells in immunocompromised mice enables their propagation as tumours that retain their neuroendocrine phenotype [27]. An orthotopic mouse model, based on injection of BON-1 and QGP-1 cells into recipient mice pancreata, previously demonstrated the successful use of cyclin-dependent kinase inhibition (using ZK 307409) on tumour progression and angiogenesis in both primary and metastatic stages [28]. Similarly, the importance of the sumoylation-enhancing protein RSUME in stabilising the tumour suppressor gene PTEN was highlighted in a study by Wu et al. which found that RSUME-null BON-1 cells implanted in nude mice showed accelerated tumour progression subsequent to loss of PTEN expression [29].

### 2.3. Primary pNET Cell Cultures

Recognising the above discussed shortcomings of cell lines and their questionable predictive value following modification while in long-term culture, the use of primary cells taken from biopsies obtained surgically or by endoscopic sampling has gained popularity among researchers. The culture of primary pNET cells taken from 25 tumours in bovine ECM has been described by Mohamed et al. [30]. This newly described culture method was used to test the effects of combination treatments using everolimus and two different somatostatin analogues (octreotide and pasireotide) on Akt upregulation and serotonin receptor type 2 trafficking. In this work however, this combination of treatments did not prove to be beneficial [31]. A similar use of primary cells reported by Falletta et al. demonstrated a positive response to a different treatment using primary patient-derived pNET cells in drug screening. In this study, the authors reported the successful development of 16 pNET primary cell cultures and their use to test the effects of everolimus together with IGF1 pathway blockade [32]. The rarity of pNETs and technical difficulties around tissue acquisition and successful propagation challenge the use of primary cell cultures.

## 3. Patient-Derived Xenograft (PDX) Models

PDX models require tumour resection or sampling followed by their transplantation into animals for expansion (most commonly immunodeficient mice), and they offer the advantage of preserving the original histological and genetic tumour properties as opposed to the altered genomic landscape of established cell lines [33,34]. Cancer cell in vitro response to drugs (as well as cancer cell expression of emerging biomarkers of resistance and response to therapy) can be used in conjunction with paralleled testing directly in those patients from which these cells were taken to inform personalised treatment [35] in approaches termed co-clinical trials [33]. The predictive value of PDX models in drug screening prior to clinical trials has been demonstrated in several cancer types including colorectal, lung, breast and renal cancers, as well as in pancreatic ductal adenocarcinoma (PDAC) [33,36]. The ability of PDX models to predict the failure of mTOR and SRC inhibitors (sirolimus and saracatinib, respectively) in the treatment of PDAC in clinical trials has also been demonstrated [37,38]. PDX models of neuroendocrine tumours are however scarce and only a few have been isolated from GEP-NENs [39]. Yang et al. reported their experience with PDX models of GEP-NETs, using tissue obtained from both primary and metastatic sites in 106 patients [34]. These included NETs from pancreatic, gallbladder, intestinal and rectal origins. This study highlights the difficulties associated with the development of such models of neuroendocrine tumours, as only around 10% of tumours were successfully engrafted in mice and only one (gall-bladder origin) out of these was successfully expanded. The first pNET PDX model was reported in 2018 by Chamberlain et al. This was isolated and expanded using tissue obtained from a patient with metastatic insulinoma to the liver, and it was used to test the antitumour effect of the mTOR inhibitor sapanisertib in everolimus-resistant tumours [40].

PDX models enable the study of cancer biology in a natural microenvironment, reflecting the tumour heterogenic nature [41]. However, they are technically challenging and largely overlook the intact human immune component or the human tumour microenvironment. Successful engraftment rates are often low for certain tumours, especially when only a limited amount of implantable tissue has been isolated [41], as demonstrated by Yang et al. within the context of modelling neuroendocrine tumours [34].

## 4. Genetically Engineered Mouse Models (GEMMs) of pNETs

**Figure 1:**
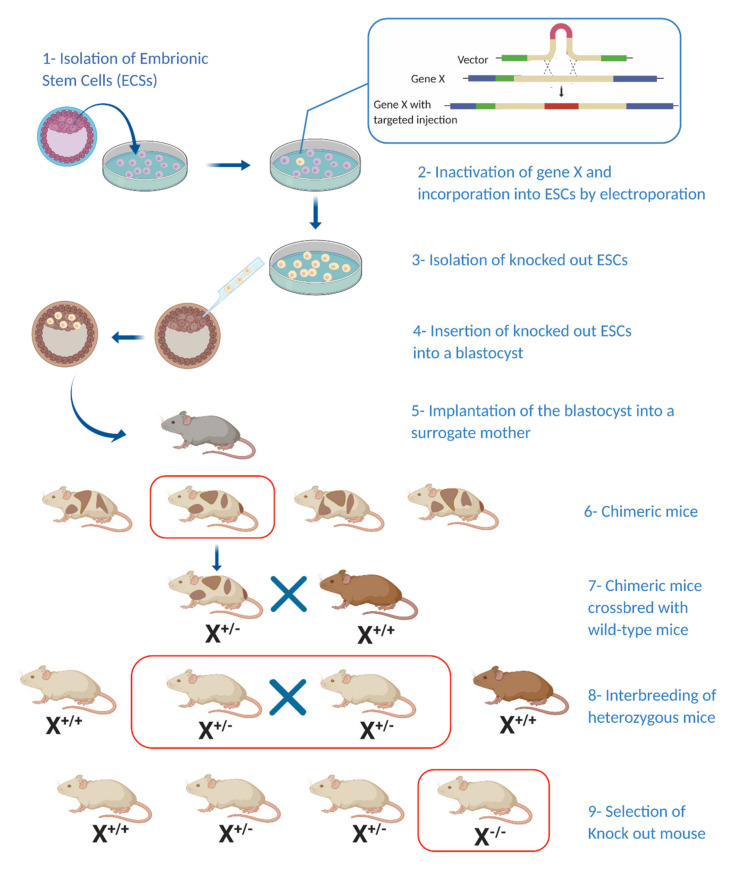
Generating a knockout mouse model: Generation of a knockout mouse model involves the incorporation of a predesigned target gene into embryonic stem cells (ESCs) (**1–3**) followed by their incorporation into a blastocyst and later into a surrogate mouse (**4–5**). Chimeric offspring are crossbred with wild-type mice and new-born heterozygous mice are bred again to produce homozygous offspring bearing the mutation of interest (**6–9**). (X^*/*^—Hetero/homozygosity for target gene) (Illustration created with BioRender.com.)

Mouse models provide a productive platform to study pNETs as they allow simultaneous observation of small populations, which have well-characterised genotypes and a short life span, alongside the ease with which malignant lesions and their precursors can be retrieved. Moreover, advances in genetics and gene manipulation allow relatively simple induction of tumourigenesis [42]. The rarity of pNETs—their complex functionality and hormone production and the difficulties associated with obtaining patient tissue samples—have made animal models an attractive alternative way to study these tumours. Current genetically engineered mouse models (GEMMs) for studying NETs (Figure 1) are mostly based on one of three approaches: those made using tissue-specific, transgenic expression of certain oncogenes (typically under the insulin or pre-proglucagon promoter); gene-knockout mice (such as the MEN-1 mouse model); and murine models that develop pNETs as part of other pathological processes.

### 4.1. GEMMs of Insulinomas

The RIP-Tag2 and RIP-Tag5 models are examples of genetic alteration in which the tissue-specific, transgenic expression of the oncogenic Simian virus 40 large T-antigen is directed by the rat insulin gene-2 promoter (RIP) [43,44]. The insulin promoter (Insm1) is highly active in pancreatic ß-cells and therefore can be used to induce tumourigenesis as early as embryonic stages. ß-cell hyperplasia and subsequent tumour formation (insulinomas) are evident several weeks into the life of these mice and are characterised by an aggressive phenotype [43]. Interestingly, low expression of Insm1 in RT-2 AB6F1 mice leads to the formation of larger, more invasive and metastatic nonfunctioning tumours, compared to the more differentiated insulinomas in RT-2 B6 models, that express higher levels of Insm1 [45]. Furthermore, knockout of the Insm1 gene in three different pNET cell lines (BON1, QPG1 and CM) produced dedifferentiated and more invasive cells in transwell three-dimensional cultures [45]. Finally, mice injected with cell lines knocked out for Insm1 were more likely to develop metastases than those injected with Insm-1-expressing cells. These different lines of evidence suggest that Insm1 plays an important role as a tumour-suppressor gene for NfpNETs and that differences in its level of expression can direct β-cell transformation toward either a differentiated insulinoma or a metastatic and more aggressive nonfunctioning form. The utility of Insm1 as a diagnostic marker for neuroendocrine neoplasms has been described by several authors in different organ systems (pulmonary, thyroid, pituitary, uterine and others) [46,47]. In pancreatic solid tumours, Tanigawa et al. demonstrated that Insm1 can not only improve the efficacy of chromogranin A and synaptophysin in diagnosing pNETs, but also differentiate it from PDAC [48]. A more recent study comparing its role in GEP-NENs with commonly used diagnostic markers (chromogranin A, synaptophysin and CD56) by McHugh et al. supports the use of Insm1 for the diagnosis of such neoplasms, albeit with lower sensitivities yet higher specificities compared to synaptophysin, chromogranin A and CD56 (80.9% vs. 99.1%, 88% and 95.3% and 95.7% vs. 86%, 87.3% and 86%, respectively) [46].

RIP-Tag5 mice develop tumours at a later stage, when the host immune response to these lesions can be studied [44]. Similarly, the RIP-myrAKT transgenic mouse model induces insulinoma formation by upregulation of the AKT/mTOR pathway [49]. In the pIns-c-MycER^TAM^/RIP-Bcl-x_L_-RIP models, transgenic stimulation and inhibition of the c-Myc oncogene and the apoptosis inhibitor Bcl-x_L_, respectively, promote the formation of an aggressive insulinoma subtype [50].

### 4.2. GEMMs of Glucagonomas

Glucagon-producing tumours can be studied using GLU-Tag2 transgenic mice which form tumours not only in the pancreas, but also in the brain [51]. The GLU-Tag2 model, however, is mostly used for studying intestinal NETs, as they are the predominant type of neoplasms in these mice [52].

Several GEMMs that were originally designed to study other disease processes but surprisingly were found to develop pNETs have been reported. These include the Gcgr^−/−^ model, in which glucagon receptor deletion is used to study diabetes-associated glucagon signalling and the oncogenic effects of deletion of the p53 and Rb tumour-suppressor genes in renin-expressing cells. These mice harbour renal and subcutaneous tumours together with glucagonomas [53] alongside other types of tumours. Glucagonomas formed in Gcgr^−/−^ mice usually remain localised, and hence the process of metastasis cannot be studied adequately in this model. To overcome this deficiency, the Gcg gfp/gfp mouse model (GCGKO), homozygous for glucagon-knockout/green fluorescent protein knock-in alleles, was developed [54]. Gcg gfp/gfp mice develop α-cell hyperplasia similarly to Gcgr^−/−^ models but also develop more invasive metastatic neuroendocrine tumours. The difference between the two models might be explained by the absence of GLP-1 expression in Gcg gfp/gfp mice, owing to its assumed role in inhibition of α-cell proliferation and subsequent hyperplasia [54]. Further studies, however, are required to determine the role of GLP in pNET tumourigenesis. GCGKO mice are therefore better mouse models than Gcgr^−/−^ for studying dissemination and metastasis in pNETs.

### 4.3. GEMMs of Non-Functioning pNETs (NfpNETs)

The NfpNET GEMM (*Prkar1a*^fl/fl^ and *Pdx1*-CRE) is characterised by inactivation of the PRKAR1A gene, a regulator of the cAMP-dependent kinase PKA [55,56]. Inactivating mutations resulting in PKA dysfunction lead to increased phosphorylation of downstream elements with resultant aberrations in cell cycle progression and apoptosis [55]. The function of PRKAR1A as a tumour suppressor gene and its role in tumourigenesis following loss of function have also been established in certain endocrine syndromes such as Carney complex multiple endocrine neoplasia. Carney complex syndrome has been linked with a predisposition to pancreatic endocrine tumours [55]. Mice with null alleles of this gene developed mixed NfpNETs with an acinar component in 100% of cases. Thus, PRKAR1A may represent a second pancreatic tumour-suppressor gene, and this model promises to enable studies of the efficacy of targeting the PKA pathway in NfpNETs.

### 4.4. GEMMs of Multiple Endocrine Neoplasia 1 (MEN-1)

The in vivo study of MEN-1-associated pNETs is possible using MEN-1-knockout mice [42]. The development of synchronous pituitary and parathyroid tumours together with pNETs allows recapitulation of the syndrome and provides platform to study the effects of different anti-neoplastic agents. Islet-cell-specific biallelic deletion of the MEN-1 gene can be achieved using the Cre-Lox method. This method can be used to induce insulinoma formation in RIP-Cre models. In some models including α-cell-specific Cre-Lox-mediated deletions, however, development of variable islet cell tumours rather than a specific histological subtype has been observed [57]. In addition, the fact that mice develop tumours early in the embryonic stage precludes their use in the study of early events leading to tumour formation in adults. A recent solution to these issues was reported by Lines et al., who generated a β-cell specific, temporally controlled mouse model (*MEN-1^L/L^*/*RIP2-CreER*). This model was developed by crossbreeding MEN-1 floxed mice (MEN-1^L/L^) carrying a tamoxifen-inducible Cre recombinase with mice expressing a modified oestrogen receptor [42]. Wong et al. [58] subsequently described two new GEMMs—MPR (MEN-1^flox/flox^ Pten^flox/flox^ RIP-Cre) and MPM (MEN-1^flox/flox^ Pten^flox/flox^ MIP-Cre)—characterised by loss of both MEN-1 and PTEN1 using the Cre-LoxP system. Both models developed well-differentiated insulinomas faster than control models with a single MEN-1 gene inactivation. Even though mice with a single PTEN gene loss do not develop pNETs, this gene may cooperate with MEN-1 to suppress pNET tumourigenesis. Consistent with PTEN deletion causing an aberrant activation of the mTOR pathway, treatment with the mTOR inhibitor rapamycin induced delayed tumour growth in these models.

## 5. 3D In Vitro Disease Models

The use of 3D in vitro models has gained popularity in recent years under the assumption that these better recapitulate the complex cell-to-cell interactions that exist in tumours. Despite their reduced capture of certain features of the physiological microenvironment (vasculature, matrix composition/stiffness, etc.) that detrimentally alter cancer cell phenotypic properties [59,60], a 3D configuration, the ability to co-culture different cell types (cancer cells, endothelial cells, fibroblasts, immune cells, etc.), the control of hypoxic conditions as found in vivo and study of drug diffusion kinetics are enabled by the more realistic tumour spatial configuration.

3D cultures also allow control over most experimental variables compared to in vivo animal models and rely on the induction of cellular aggregate (tumour spheroid) formation that can be incorporated into or grown on top of an extracellular substitute, acting as miniature 3D models of the tumour and its immediate environment [59]. Moreover, high-throughput drug screening requires a technically simple and highly reproducible platform that can be expanded within a short time period which can suitably be done using such cultures [61].

### 5.1. Spheroid-Based 3D Culture Models

Spheroids are 3D culture models that are grown under nonadherent conditions, with their use going back to the early 1970s [62]. Tumour spheroids are essentially self-organising spherical aggregates that arise from cell suspensions of cell lines, PDXs or stem cells which can also be co-cultured with other stromal components (i.e., endothelial, mesenchymal and immune cells) [63]. With complex cell–cell and cell–matrix interaction owing to their 3D spatial arrangement, these structures allow the formation of nutrient, gas and growth factor gradients that more realistically mimic in vivo conditions in comparison with monolayer cultures [64]. The formation of spheroids is assumed to occur due to changes in cell adhesion and differentiation patterns and is believed to occur in a stepwise manner. Firstly, single cells use cell surface integrin mediated interaction with the ECM to form direct cell–cell contacts with resulting increased expression of cadherin molecules. Increased cell membrane cadherin expression on different cells in turn allows these cells to bind and form compact cell clusters [65].

**Figure 2:**
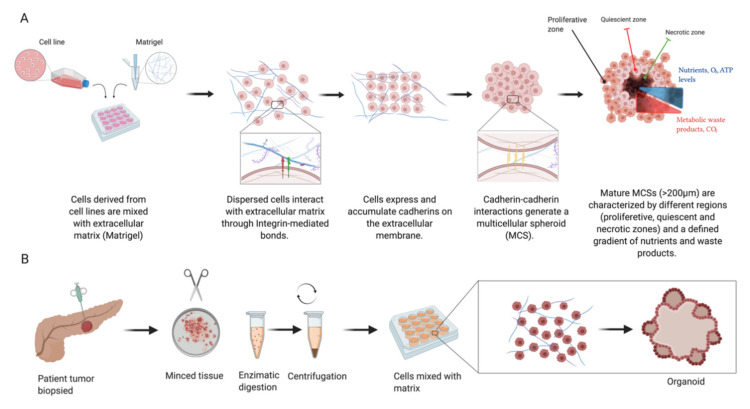
Multicellular spheroids (MCSs) are derived from cell lines or patient-derived xenograft (PDX) cells (**A**). Increased cellular expression of cadherins and their intercellular cadherin–cadherin interactions lead to spheroid compaction. Mature spheroids show a concentric organisation into multilayered structures leading to nutrient gradient formation, resulting in insufficient transport towards their core as well as elimination of waste products. Organoids (**B**) are derived from primary tissue stem cells and their culture gives rise to structures mimicking the original tissue architecture. (Illustration created with BioRender.com.)

Cellular spheroids are concentrically arranged into several layers (Figure 2)—an external proliferating and metabolically active peripheral layer, a middle quiescent cell layer and a central necrotic core (more evident in spheroids >200 μm in diameter) [66]. Due to the limited diffusion capacity (150–200 μm) of oxygen and nutrients, spheroids show internal features of different oxygen tension (leading to hypoxic conditions at their core), metabolite accumulation and different proliferation patterns [63]. This specific feature is one of many advantages that these sorts of models offer over traditional 2D monolayer cultures, in the sense that this process closely resembles biological processes observed in cancers in vivo. A known feature of cancers is the poorly organised vasculature and leakage resulting from aberrant vasculogenesis which leads to inadequate nutrient supply. Importantly, the imbalance between tumour cell expenditure and functional feeding vessels produces central tumour necrosis due to the distance (and hence reduced nutrient supply) from body vasculature. This known property of cancers has a detrimental effect on their response to different chemotherapy agents and radiotherapy. Therefore, the existence of this phenomenon in a model used for drug screening or studying the biology of cancer makes such a model more relevant compared to monolayer cultures [66,67,68].

Spheroid models of pancreatic ductal adenocarcinomas have previously demonstrated higher chemoresistance compared to conventional monolayer platforms using the MiaPaca2 and PANC-1 cell lines [69]. The use of a pancreatic cell line (PANC-1) together with stromal cells for studying the interaction of cancer cells with the ECM as well as their combined effect on treatment response to doxorubicin, gemcitabine and paclitaxel has been reported recently [64,70]. Reports of pNET 3D models are relatively scarce; however, early work focusing on pNET spheroid culture models and their characterisation has been reported. In a recent study published by Bresciani et al., the BON-1 pNET cell line was used to compare three culture methods, with ultralow-attachment 96-well culture plates (ULA) showing superior results in terms of technical ease and experimental reproducibility, while investigating the effect of the tyrosine kinase receptor inhibitor sunitinib [71]. In another study, the βTC3 murine pancreatic β-cell line was used to study the effects of stimulation of the platelet-derived growth factor (PDGF) signalling cascade based on overexpression of the PDGF receptor β. Cells subject to PDGF-DD stimulation showed enhanced functional tumour heterogeneity in spheroids grown in ultralow-attachment plates (Corning Life Sciences) [72]. Lastly, spheroid cultures of the pNET cell lines BON-1 and QGP-1 showed higher viability in serum-deprived culture conditions, while demonstrating more realistic tumour features such as more physiological levels of characteristic pNET markers, somatostatin and dopamine receptor expression changes (following targeted treatment) and treatment resistance when compared to monolayer cultures. This recent study also supports the use of spheroid models in proteomics-based searches for biomarkers, as under these approaches, serum starvation before protein analysis is commonly performed to avoid serum protein contamination [73].

### 5.2. Organoid Culture Models of pNETs

Organoid and spheroid cultures have gained significant popularity in recent years as disease models for the study of organ development, tumour biology, drug screening and toxicity [74]. The terms spheroids and organoids are often used interchangeably; however, they are in fact different entities that, by definition, vary in their cells of origin and biological properties. Both organoid and spheroid cultures offer advantages and disadvantages over each other, and the choosing of one specific approach should be goal-oriented (Table 1). Organoids can be defined as a “collection of organ-specific cell types that develops from stem cells (ESCs or PSCs) or organ progenitors and self-organise through cell sorting and spatially restricted lineage commitment in a manner similar to in vivo” [75]. Organoid cultures are derived from tissue biopsies and preserve the functionality of their tissue of origin [76]. Such 3D models have been developed from several organ systems, allowing better understanding of the process of organogenesis. For the purpose of cancer research, they are most often grown in hydrogels, with the murine sarcoma basement membrane based Matrigel (Corning) being particularly popular for cancer cultures [61,77,78,79]. In many tumour types, organoids have been found to more closely recapitulate the in vivo properties of tumours compared to 2D cultures of immortalised cell lines. The ability to culture patient-derived cancer cells allows more accurate understanding of individual tumour behaviour alongside assessment of patient-specific treatment strategies. Organoids have been developed from gastrointestinal cancers arising in various tissues [80], including PDAC, with 75–85% success rates [81]. Organoid-based cultures of pNETs, however, remain underdeveloped. April-Monn et al. recently described the long-term culture and use of primary pNET cells which grew to form islet-like tumouroids as a drug screening platform for treatments such as sunitinib, everolimus and temozolomide. This report emphasised how drug response varies between patients and demonstrated the platform’s ability to be potentially used as a predictive instrument in larger cohorts [82].

## 6. The Tumour Microenvironment Is an Important Feature of the Disease Process as a Whole

Despite their advantage over traditional monolayer cultures, spheroid and organoid cultures neglect the role of the tumour microenvironment in disease progression. The ECM is a complex network of structural and signalling protein, also consisting of proteoglycans which serve not only as a support framework in different tissues but also as significant participants in mechanical and biochemical signalling mediated by cell surface receptors [91,92]. ECM proteins bear domains that can bind cell-matrix adhesion receptors such as integrins as well as those that have dedicated roles in cellular signal transduction and growth-factor-mediated processes including angiogenesis. Other tissue remodelling processes such as degradation and deposition of cancer-associated ECM proteins by cancer and cancer-supporting cells are similarly pivotal for disease progression.

Noncancerous supporting cells promote cancer cell survival and tumour structure and function, and they are associated with the chemoresistance that challenges effective cancer treatment [93].

Cancer-associated stromal cells are also able to modulate the antitumour immune response by interacting with immune cells by secreting chemokines that have an immunosuppressive as well as proapoptotic role against T-cells [94].

### 6.1. Modelling the pNET Microenvironment

The use of animal and 3D culture models has enabled researchers not only to study genetic aberrations leading to pNET formation and the efficacy of novel therapeutic agents, but also to study the close interaction between tumour cells and their microenvironment. In vitro cancer disease models have also been significantly modified to include both artificial and natural ECM components, as well as a variety of cancer-supporting cells, in attempts to study cancer development, progression and response to treatment under more realistic biological conditions.

#### 6.1.1. The Role of the ECM in pNET Progression

Studies looking into the close interaction between NETs and their stroma have revealed that NETs are one of the highly vascularised cancers and are 10-fold more vascularised compared to epithelial-cell-derived carcinomas [95]. NET cells highly express proangiogenic factors (VEGF, PDGF, FGF, angiopoietins) that induce not only recruitment but also proliferation of vessel progenitor cells. Activation of cancer-associated fibroblasts (CAFs) occurs both locally and at more distant foci under paracrine control using secreted serotonin, TGF-β and connective tissue growth factors and reciprocally promotes tumour progression and ECM remodelling by the release of profibrotic factors. Comparisons of healthy pancreatic islets with their neoplastic counterparts revealed alterations in ECM composition (expression of fibrinogens, lectins, galectin-1, laminins and other glycoproteins) which were also dependent on histological grade [95,96]. Several ECM glycoproteins have been found to play a role in both early and late stages of tumourigenesis. Tenascin-C, for example, was shown to be an activator of the WNT pathway and hence to promote tumour survival, proliferation, angiogenesis and progression, as observed in a study performed in a RIP1-Tag2 (RT2) mouse model [97]. The process of ECM remodelling involves degradation and biosynthesis of structural proteins. ECM degradation is assumed to be a function of two important protease families—heparanases and MMPs. Heparanases cleave heparan sulphate chains on ECM glycoproteins and thereby relax the ECM structure and also release bound growth factors [91]. Heparanase expression levels in pNETs have shown to correlate with disease stage and distant spread. This observation was reported in a study performed on an RT2 mouse model using heparanase-transgenic mice (*Hpa*-Tg), which constitutively overexpress heparinase, and heparanase-knockout mice (*Hpse^−/−^*) [98].

#### 6.1.2. Tissue Martrikines Modulate Disease Processes

Products of ECM degradation produced by proteolytic activity of enzymes such as MMPs are collectively called matrikines [99]. Modulation of certain processes such as proliferation, migration and apoptosis have been previously linked with matrikines, highlighting their key role in the control of tumour progression [99]. MMP2 and -9 are proteinases with a range of substrate specificities that have shown to be upregulated in GEP-NETs (and are detectable in bodily fluids of patients with NETs) [95]. MMP9 has been implicated in the activation of angiogenesis in pNETs by its catalysis of the proteolytic release of ECM-bound VEGF [95,100]. The upregulation of MMP2 in pancreatic cancer has been established as an independent predictor of pNETs, while the levels of endogenous tissue inhibitors of matrix metalloprotease (TIMP) can differentiate between PDAC and pNETs [101]. The roles of other matrikines in the progression of pNETs have also been described following observations made in in vivo models, most commonly of murine origin. The RT2 model has been used to study the role of fibronectin and its biologically active degradation products as well as the administration of other angiogenic regulatory factors (endostatin, thrombospondin-1 and tumstatin) on tumour progression and survival [102,103,104]. Neprilysin is an endopeptidase that is overexpressed in NETs with a background of MEN-1/DAXX mutations and has also been linked to the promotion of proliferation of both benign and malignant β-cell neoplasms by its modulation of the action of certain regulatory peptides [105,106]. Feng et al. showed that viral transfection using specific short hairpin RNA (shRNA) sequences targeting the neprilysin gene in insulin-secreting β-cells (rat INS-1 cells) and its subsequent knockdown reduced tumour formation when these were implanted in athymic nude mice [106].

### 6.2. Native Tissue Constructs as Potential Alternatives to Animal Models

Despite the overall benefit gained so far, the use of animal models raises ethical issues around subjecting animals to discomfort and death. Moreover, such models are limited in their ability to represent complex biological processes around cancer physiology, development and progression in humans due to interspecies differences in genetics and biology, as well as variability in their expression of molecular targets [107]. Alongside ethical issues, lengthy and complex protocols as well as high costs involved in breeding and maintenance are additional disadvantages that should be considered. Moreover, successful translations from animal to human clinical trials are often as low as 8% [107,108]. Considering the above, the use of animals has been an area of debate, and alternatives to animal experimentation have been proposed to overcome the above difficulties and disadvantages of working with animal models. A reduction, refinement and replacement (the 3R) approach is currently being applied in preclinical research studies, aiming to reduce unnecessary suffering and use of animals where reasonable alternatives are available [109]. The rapidly developing field of tissue engineering allows researchers to use alternative platforms of both artificial and natural tissue constructs to more realistically mimic complex biological environments. One such appealing approach includes modelling disease processes in native tissue scaffolds derived from human organ donors, which are first decellularised in order to isolate the ECM. Isolation of native ECM allows the use of site-specific proteins while also providing protein footprints of past cellular inhabitants. The process of decellularisation typically involves physical (e.g., freezing and thawing), detergent (e.g., Triton-X100, SDS) or enzymatic (e.g., trypsin) removal of resident cells without gross disruption of the architecture and biological properties of the ECM [110,111]. Devoid of their previous cellular inhabitants, these constructs are later repopulated with cells of interest in order to study cell–ECM interactions.

#### Tissue Decellularisation Enables Profiling of the Cancer Matrisome

Advances in proteomics, more specifically ‘bottom-up’ proteomics where samples are enzymatically digested into peptides prior to analysis using mass spectrometry, enable quantitative profiling of the complex in vivo ECM protein composition [112]. Differences in expression of structural and functional proteins result in unique ECM-protein signatures that can be used to differentiate between normal and (pre)cancerous tissue, allowing for identification of changes in the tumour microenvironment prior to significant progression and metastasis. Such changes were observed in a range of cancer types including pancreatic, colonic, ovarian, liver and haematological malignancies [113,114,115,116]. A key step (known as ‘the angiogenic switch’) in cancer progression is the stage at which tumours become highly vascularised. Using a mouse model of insulinoma (B6.D2-Tg(RIP1-Tag2)2Dh (RIP1-Tag2)), Naba et al. applied advanced quantitative proteomics (isobaric tag labelling (iTRAQ)) to profile the decellularised ECM of normal, hyperplastic, and angiogenic islets, as well as that of insulinomas, and demonstrated a higher abundance of 9 and lower abundance of 26 ECM proteins that correlated with insulinoma progression [117].

Tissue decellularisation can also be applied to construct platforms for in vitro 3D disease modelling of cancers within their native tissue. When designing culture models, it is not only the ability to combine known components of the ECM, such as collagens, fibronectin, proteoglycans and growth factors, that is key. The association, concentrations and ratios of ECM components play an important role in preserving its function and unique characteristics that will support cellular processes, growth and function [118,119]. The role of pancreatic ECM in mediating pancreatic islet and β-cell differentiation, growth, function and survival has also been described [120]. The preservation of tissue architecture and biochemical properties using whole organ decellularisation and subsequent recellularisation using healthy or cancer cells allows the construction of experimental models for tissue regeneration and ex vivo modelling of cancers, respectively [110]. Whole organ decellularisation of kidney, liver, heart and lungs has been reported in both murine and human organs [121]. Successful decellularisation of murine [122,123] and human pancreata [119,120] has been described with the aim of developing a platform for β-cell islet transplantation and the study of islet organogenesis [124,125]. Although reports of decellularised-tissue-based models of several cancer types including liver, colon, lung, brain and breast exist [126,127,128,129], pancreatic cancer models of this type are lacking. Such models could potentially provide novel means for ex vivo disease modelling to study the close interaction of islet cell cancers and their immediate microenvironment, within their native tissue rather than in cross-species platforms.

## 7. Summary

Neuroendocrine tumours of the pancreas are a rare entity, yet their incidence is on the rise. The often poor outcomes associated with pNETs, however, warrant improvement in their management and therefore support further research of the disease. Disease models are pivotal in ex vivo studies of biological and molecular pathogenetic processes in cancer and have evolved over the last few decades, with technological advances allowing the development of more complex platforms.

Traditional 2D monolayer cultures of established cell lines were key in preclinical drug development stages that resulted in approval of current first-line agents for treatment of pNETs such as sunitinib. Such models, however, are insufficient for studying more complex genetic characteristics and overlook the in vivo like effects of the tumour environment on disease progression and chemoresistance. Moreover, they do not align with the current shift in research toward a more personalised treatment approach. Models that consider the in vivo like cell–cell interactions and spatial arrangement, such as spheroids, allow for more realistic studies preserving to some extent oxygen and nutrient gradients that are observed in malignant masses. With respect to neuroendocrine cancer of pancreatic origin, reports of spheroid cultures have mostly focused on model characterisation while observing specific cell processes, investigating molecular signalling (i.e., PDGF signalling cascade) and conducting in vitro drug screening.

Organoids that are derived directly from patient biopsies more closely resemble the tumour of origin than 2D cell lines and maintain genetic expression and aberrations while preserving the tumour architecture yet are technically more challenging. Despite a recent trend toward organoid-based research in other cancer types, reports of NET-derived organoids are lacking. This is mostly attributed to the rarity of the disease and the technical challenges (including slow tumour growth rates) involved in culture of pNET primary cells. Since organoids have been used for the study of embryonic tissue development, the study of the role of cancer stem cells, as well as cancer-specific processes, is also likely to prove valuable for studying the development and progression of NETs. A very recent study by April-Monn et al. highlighted the potential of organoid models as a personalised drug screening approach performed on primary pNET cultures [82].

The ability to artificially induce known associated genetic aberrations which lead to tumour formation made GEMMs an attractive platform for studying pNETs (and other cancers in general), which have been extensively linked with genetic syndromes and various signalling pathway alterations. Transgenic mouse models in which MEN-1 and PTEN gene knockouts are induced (resembling mutations identified in a subset of pNETs) are therefore extremely valuable. Animal models also enable the study of cancer cell–ECM interactions, as tumours are observed within their supporting tissue. However, the low clinical translatability rates of findings in animals could perhaps be improved by using tissue constructs obtained from surgical resections and from transplant and deceased donors. Decellularised tissue depleted of resident cells retains the ECM components that confer important features such as physiological substrate stiffness and protein signatures of the native tissue. In the context of studies involving the pancreatic endocrine system, such existing models mostly focus on tissue regeneration and islet transplantation for the treatment of diabetes. The combination of such tissue constructs with primary patient cancer cells has the potential to provide a more accurate ex vivo platform to study cancer-induced ECM remodelling.

## Figures and Tables

**Table 1:** Organoid and spheroid cultures offer advantages and disadvantages over each other, and the choice of a specific approach should be goal-oriented.

	Cells of Origin	Biological Properties	Advantages and Potential Use	Disadvantages
**Spheroids**	Cell lines [83,84,85] PDX cells [86]	Cultured in suspension in basement membrane elements Spheroid size and compactness vary between cell lines [85] Mimic cell–cell interactions that promote proliferation and survival observed in vivo [86] Trilaminar structure (Proliferative, quiescent, and necrotic zones) [85] Dynamic growth rate similar to those observed in vivo: early exponential phase with cell proliferation followed by a decline in growth with increased necrosis [85]	Recapitulates mismatch between uncontrolled growth and nutrient supply [85] Hypoxic environment in spheroid core promotes evolution of cancer stem cells [86] The use of co-cultures with stromal components enables studying signalling pathways, angiogenesis and invasion [85] Ideal for high-throughput drug screening in more in vivo like spatial arrangement of cells compared to monolayer cultures [86]	Limited clinical value due to the absence of biological complexity and functionality of in vivo tumours [83]
**Organoids**	Cells derived from dissociated primary tissue [83,86] Stem cells [87] Genetic manipulation of cells derived from normal tissues [88,89]	Cultured in suspension in basement membrane elements Self-organising or organogenesis-cue-dependent Reproduce functionality and architecture of the tissue of origin [86] Preservation of genetic expression and histopathologic features of the original tumour [86]	Offer a tumour-specific and personalised approach to treatment [86] Orthotopic transplantation of organoids in mice reproduces primary tumour properties [36] Creation of organoid biobanks of tumours derived from patients with different tumour phenotypes [36] Study the role of infectious pathogens in tumourigenesis [36] Modelling of cancer associated genetic aberrations using CRISPR/Cas9 genome editing [88] Study the effect of drugs in a way more similar to the conditions in vivo compared to 2D models [86]	Risk of genetic drift from original tumour due to instability of tumour genome. This process can be reduced by the restriction of culture time [90] Genetically engineered organoids show lower metastatic potential compared to those derived from primary tumours [88] Lack vasculature and immune components [36,86]

## Supplementary Materials

The following are available online at https://www.mdpi.com/2072-6694/12/11/3170/s1, Figure S1: Currently available disease models of pNETs: cell lines, patient-derived xenograft (PDX), animal models (GEMMs), spheroids and organoids.
