# Supplementary material for: Modelling Pancreatic Neuroendocrine Cancer: From Bench Side to Clinic
Source: Cancers (Basel). 2020 Oct 28;12(11):3170. doi: 10.3390/cancers12113170 (PMC7693644; doi:10.3390/cancers12113170)
Supplement: Supplementary file 1 [file cancers-12-03170-s001.pdf]

Supplementary Materials: Stage 1: Modelling Pancreatic Neuroendocrine Cancer: From Bench Side to Clinic

Alexander Ney, Gabriele Canciani, J. Justin Hsuan and Stephen P. Pereira

Supplementary Table S1. Currently available disease models of pNETs: cell lines, patient-derived xenograft (PDX), animal models (GEMMs), spheroids and organoids.

	Models	Features	Used For	Outcome and Translation	References
Cell Lines	BON-1	Derived from metastatic lymph node of human pancreatic serotonin producing NET. P53, TSC2 and NRAS mutations with aberrant activation of mTOR/Akt pathway	Test tacrolimus and somatostatin-analogue (SSA) treatment	Antisecretory treatment for NETs. As monotherapy or in combination with antiproliferative therapy of neuroendocrine tumours currently in high-quality phase III trials	[1,3,4]
	QPG-1	Cells derived from human primary pancreatic NET. P53, ATRX and SMAD4 mutations	Test tacrolimus and somatostatin-analogue treatment	Antisecretory treatment for NETs. As monotherapy or in combination with antiproliferative management of neuroendocrine tumours currently in high-quality phase III trials	[2,3,4]
	BON-1 and QPG-1 orthotopic mouse model	BON-1 and QPG1 injected in recipient mice pancreas	Study the effects of cyclin-dependent kinase inhibition on tumour progression	ZK304709-treated cells showed increased propensity for apoptosis and reduction in angiogenesis	[5]
	RSUME knockdown BON-1 cells	RSUME knockdown BON-1 cells,	Study tumour progression after RSUME	pNETs with downregulation of RSUME grew faster	[6]

	implanted in nude mice	downregulation—a stabilising factor of PTEN	with increased liver metastases compared to those with normal RSUME expression	
CM	Cells derived from ascitic fluid of a human patient with primary pancreatic NET	Investigate whether insulinoma CM cells retain β-cell function. CM cells have a functional glucose-signalling pathway and insulin mRNA expression similar to normal β-cells	The use of gefitinib (EGFR-TK inhibitor) treatment resulted in growth inhibition, apoptosis and cell-cycle arrest in CM cells	[7,8]
NT-3	Cells derived from metastatic lymph node of human pancreatic serotonin producing NET	Develop a well-differentiated and high SSTR-expressing tumour model	Octreotide, everolimus and streptozocin showed better response in NT-3 cells than in low SSTR-expressing QGP-1 cells	[9]
Primary pNET cells in bovine ECM	Human primary pancreatic NET cells derived from 15 patients in a bovine extracellular matrix culture	Test the effects of everolimus and somatostatin analogues	No benefit in everolimus and SSA combined treatment. Caspase-dependent apoptosis induced by SSA is reduced when administered in combination with everolimus	[10,11]
Primary pNET cells in bovine ECM	Human primary pancreatic NET cells derived from 16 patients (18 primary pNets and 2 metastases) cultured in bovine	Search for possible surrogate markers of response to everolimus	In vitro responsiveness to everolimus relates to in vivo efficacy in patients. pNET primary cultures could be used as a model to predict everolimus efficacy	[12]

		extracellular matrix culture			
PDXs:	Primary pNET cells	58 pancreatic, 1 gallbladder, 38 small bowel and 3 rectal tumours.	Develop a NET xenograft model	7 were successfully engrafted (3 pancreatic, 3 of intestinal and 1 of gallbladder origin) of which only 1 was propagated for 8 passages (gallbladder origin)	[13]
	PDX-pNET	Cells derived from a metastatic insulin producing NET	Test the effects of sapanisertib in everolimus resistant tumours	Sapanisertib treatment induced tumour volume reduction in everolimus resistant tumours	[14]
Animal Models (GEMMs)	RIP-Tag2<br>RIP-Tag5	GEMM SV40 large T-antigen directed oncogenic transcription of the rat insulin gene-2 promoter (RIP)	To develop insulinoma model and study the host immune response to the tumour	Tag expression induces β-cells hyperplasia and tumour progression. The lymphocytic infiltration was observed in islets overexpressing L-selectin and $\alpha4\beta7$	[15,16]
	RIP-Tag2 AB6F1	RIP-Tag2 GEMMs are derived from hybridization of AB6FT genetic background and RIP-Tag2 mice. These models develop nonfunctioning pNETs	Develop nonfunctioning RIP-Tag2 tumour models and study the role of Insm1 gene in pNET differentiation	RT2 AB6F1 mice developed nonfunctioning tumours that were larger with higher metastatic capacity than those observed in RT2 B6 mice	[17]

RIP-myrAKT	GEMM overexpressing the active form of AKT1 under the rat insulin promoter	Develop an insulinoma model and study the AKT1/mTOR pathway	RIP-myrAKT mice developed β cell hyperplasia and tumours. The oncogenic transformation correlated with PTEN downregulation	[18]
pIns-c-MycER^{TAM}/RIP-Bcl-x$_L$-RIP	GEMM with transgenic stimulation of c-Myc and Bcl-x$_L$ under control of an insulin promoter	Study the combined effect of Myc and Bcl-x$_L$ stimulation on tumourigenesis	Activation of c-Myc generates proliferation of B-cells but is controlled by apoptosis. Co-suppression of c-myc and bcl-x provokes progression into angiogenic and invasive tumours. Deactivation of c-myc induces vascular degeneration and apoptosis	[19]
GLU2-Tag	Glucagon-promoted simian virus T-antigen oncogene harboring GEMM	Provide a model for human insulinoma	The mice developed differentiated tumours and showed high levels of circulating glucagon	[20,21]
Men-1$^{L/L}$/RIP2-CreER	Crossbred GEMMs with mice carrying tamoxifen-inducible Cre recombinase under the control of the rat insulin promoter	Generate a temporally controlled conditional tumour model using tamoxifen in the mice diet	GEMMS treated with tamoxifen showed increased B-cell proliferation compared to control mice. This model enables the study of early events leading to B-	[22]

			cell NET development	
Men-1LoxP/LoxP-RIPCre+	MEN-1 inactivation using Cre-Lox method	Develop an insulinoma model	Cell hyperplasia was observed in the endocrine pancreas and pituitary gland, yet only pNETs were observed	[23]
Men-1$^{flox/flox}$ Pten$^{flox/flox}$ RIP-Cre (MPR)<br><br>Men-1$^{flox/flox}$ Pten$^{flox/flox}$ MIP-Cre (MPM)	GEMMs with inactivation of both MEN-1 and PTEN	Evaluate the combination of two genes inactivation in tumourigenesis	Both models developed well-differentiated tumours faster than models with single deletion of MEN-1 or PTEN. MPR developed pituitary NETs as well. Treatment with rapamycin delayed the growth of tumours in both models	[24]
Gcgr$^{-/-}$	Gcgr with deletion of p53 and Rb in renin-expressing pancreas cells	Develop a highly penetrant and metastatic glucagonoma model	The use of fluorescent reporters allows the study of metastasis and identification of potential molecular targets	[25]
Gcg gfp/gfp (GCGKO)	Gfp knock-in GEMM	Develop a glucagonon producing tumour model and study metastases in pNETS	The pNETS in GCGKO mice developed from α-cells and disseminated in lungs and liver	[26]
Δ-Prkar1a	PRKAR1A Knockout GEMM	Study the role of PKA pathway in pNET tumourigenesis	Mice developed tumours endocrine or mixed carcinomas with 100% penetrance. Stromal and lymph	[27]

				node invasion were observed.	
	RT2 Hpa-Tg<br><br>RT2 Hpse$^{-/-}$	RT2 mouse model constitutively expresses heparinase and RT2 heparinase knockout model	Study the role of heparinase in tumour microenvironment with the observation of overexpressing and knockout mouse models	RT2 Hpa-tg mice overexpressing heparinase developed tumours with increased peritumoural lymph-angiogenesis<br><br>Heparinase deletion in RT2 Hpse$^{-/-}$ provoked increased angiogenesis and pericyte coverage	[28]
3D Cultures—Spheroids	BON1 and QPG-1 serum deprived	BON1 and QPG1 3D models	Study the effects of serum deprivation on BON1 and QPG1 spheroids and in comparison with 2D monolayer culture	In BON1 spheroids, total cell number, serotonin and chromogranin A secretion increased in parallel.<br><br>QPG1 showed the most evident changes in mRNA expression of somatostatin and D2R receptors.<br><br>Both BON1 and QPG1 spheroids showed enhanced cell survival under serum deprivation compared to 2D models	[29]

	BON-1 96-well hanging drop plates (HD plates)<br><br>BON-1 24-well plates with a cell-repellent surface<br><br>BON-1 ultralow-attachment 96-well plates (ULA plates)	Three different methods to obtain 3D culture models of BON-1 cells developing tumours under more realistic conditions compared to 2D cultures	Identification of ideal 3D culture methods for the purpose of drug screening (sunitinib)	ULA plates method proven as ideal approach in terms of reproducibility enabling single spheroid culture in each well to perform viability or cytotoxic tests	[30]
	βTC3 murine pancreatic β-cell line	Cells derived from a PDGF-DD knockout mouse model	Study tumour growth in the absence of and following stimulation of PDGF-DD	βTC3 cells stimulated by PDGF-DD developed higher number of tumour spheroids compared to untreated cells	[31]
3D Cultures—Organoids	Screening platform with islet-like tumouroids originating from primary cells of pNET patients	Tumouroids maintained the neuroendocrine phenotype of primary tumour and remained viable for at least 2 weeks with 86% of success rate	Tumouroids comparing effects of sunitinib, everolimus and temozolomide treatment	Utility of pNET organoids in drug screening confirmed as a personalised treatment approach testing, demonstrating variable responses, including between samples from the same patient	[32]

References

1. Townsend, C.M.; Ishizuka, J.; Thompson, J.C. Studies of Growth Regulation in a Neuroendocrine Cell Line. *Acta Oncol.* **1993**, *32*, 125–130, doi:10.3109/02841869309083900.
2. Kaku, M.; Nishiyama, T.; Yagawa, K.; Abe, M. Establishment of a carcinoembryonic antigen-producing cell line from human pancreatic carcinoma. *Gan* **1980**, *71(5):596-601 PMID:7227711.*
3. Stueven, A.K.; Kayser, A.; Wetz, C.; Amthauer, H.; Were, A.; Tacke, F.; Wiedenmann, B.; Roderburg, C.; Jann, H. Somatostatin Analogues in the Treatment of Neuroendocrine Tumors: Past, Present and Future. *Int. J. Mol. Sci.* **2019**, *20*, 3049.
4. Hofving, T.; Arvidsson, Y.; Almobarak. B.; Inge, L.; Pfragner, R.; Persson, M.; Stenman, G.; Kristiansson, E.; Johanson, V.; Nilsson, O. The neuroendocrine phenotype, genomic profile and therapeutic sensitivity of GEPNET cell lines. *Endocr. Relat. Cancer*. **2018**, *25*, X1–X2.
5. Scholz, A.; Wagner, K.; Welzel, M.; Remlinger, F.; Wiedenmann, B.; Siemeister, G.; Rosewicz, S.; Detjen, K.M. The oral multitarget tumour growth inhibitor, ZK 304709, inhibits growth of pancreatic neuroendocrine tumours in an orthotopic mouse model. *Gut* **2008**, *58*, 261–270, doi:10.1136/gut.2007.146415.
6. Wu, Y.; Tedesco, L.; Lucia, K.; Schlitter, A.M.; Garcia, J.M.; Esposito, I.; Auernhammer, C.J.; Theodoropoulou, M.; Arzt, E.; Renner, U.; et al. RSUME is implicated in tumorigenesis and metastasis of pancreatic neuroendocrine tumors. *Oncotarget* **2016**, *7*, 57878–57893, doi:10.18632/oncotarget.11081.
7. Baroni, M.G.; Cavallo, M.G.; Mark, M.; Monetini, L.; Stoehrer, B.; Pozzilli, P. Beta-cell gene expression and functional characterisation of the human insulinoma cell line CM. *J. Endocrinol.* **1999**, *161*, 59–68, doi:10.1677/joe.0.1610059.
8. Höpfner, M.; Sutter, A.P.; Gerst, B.; Zeitz, M.; Scherübl, H. A novel approach in the treatment of neuroendocrine gastrointestinal tumours. Targeting the epidermal growth factor receptor by gefitinib (ZD1839). *Br. J. Cancer* **2003**, *89*, 1766–1775, doi:10.1038/sj.bjc.6601346.
9. Benten, D.; Behrang, Y.; Unrau, L.; Weissmann, V.; Wolters-Eisfeld, G.; Burdak-Rothkamm, S.; Stahl, F.R.; Anlauf, M.; Grabowski, P.; Möbs, M.; et al. Establishment of the First Well-differentiated Human Pancreatic Neuroendocrine Tumor Model. *Mol. Cancer Res.* **2018**, *16*, 496–507, doi:10.1158/1541-7786.mcr-17-0163.
10. Mohamed, A.; Romano, D.; Saveanu, A.; Roche, C.; Albertelli, M.; Barbieri, F.; Brue, T.; Niccoli, P.; Delpero, J.R.; Garcia, S.; et al. Anti-proliferative and anti-secretory effects of everolimus on human pancreatic neuroendocrine tumors primary cultures: is there any benefit from combination with somatostatin analogs? *Oncotarget* **2017**, *8*, 41044–41063, doi:10.18632/oncotarget.17008.
11. Mohamed, A.; Blanchard, M.-P.; Albertelli, M.; Barbieri, F.; Brue, T.; Niccoli, P.; Delpero, J.R.; Monges, G.; Garcia, S.; Ferone, D.; et al. Pasireotide and octreotide antiproliferative effects and sst2 trafficking in human pancreatic neuroendocrine tumor cultures. *Endocrine-Related Cancer* **2014**, *21*, 691–704, doi:10.1530/erc-14-0086.
12. Falletta, S.; Partelli, S.; Rubini, C.; Nann, D.; Doria, A.; Marinoni, I.; Polenta, V.; Di Pasquale, C.; Degli Uberti, E.; Perren, A.; et al. mTOR inhibitors response and mTOR pathway in pancreatic neuroendocrine tumors. *Endocr. Relat. Cancer* **2016**, *23*, 883–891, doi:10.1530/erc-16-0329.
13. Yang, Z.; Zhang, L.; Serra, S.; Law, C.; Wei, A.; Stockley, T.L.; Ezzat, S.; Asa, S.L. Establishment and Characterization of a Human Neuroendocrine Tumor Xenograft. *Endocr. Pathol.* **2016**, *27*, 97–103, doi:10.1007/s12022-016-9429-4.
14. Chamberlain, C.E.; German, M.S.; Yang, K.; Wang, J.; VanBrocklin, H.; Regan, M.; Shokat, K.M.; Ducker, G.S.; Kim, G.E.; Hann, B.; et al. A Patient-derived Xenograft Model of Pancreatic Neuroendocrine Tumors Identifies Sapanisertib as a Possible New Treatment for Everolimus-resistant Tumors. *Mol. Cancer Ther.* **2018**, *17*, 2702–2709, doi:10.1158/1535-7163.mct-17-1204.
15. Hanahan, D. Heritable formation of pancreatic β-cell tumours in transgenic mice expressing recombinant insulin/simian virus 40 oncogenes. *Nat. Cell Biol.* **1985**, *315*, 115–122, doi:10.1038/315115a0.
16. Onrust, S.V.; Hartl, P.M.; Rosen, S.D.; Hanahan, D. Modulation of L-selectin ligand expression during an immune response accompanying tumorigenesis in transgenic mice. *J. Clin. Investig.* **1996**, *97*, 54–64, doi:10.1172/jci118406.
17. Kobayashi, S.; Contractor, T.; Vosburgh, E.; Du, Y.-C.N.; Tang, L.H.; Clausen, R.; Harris, C.R. Alleles of Insm1 determine whether RIP1-Tag2 mice produce insulinomas or nonfunctioning pancreatic neuroendocrine tumors. *Oncology* **2019**, *8*, 16, doi:10.1038/s41389-019-0127-1.

18. Alliouachene, S.; Tuttle, R.L.; Boumard, S.; Lapointe, T.; Berissi, S.; Germain, S.; Jaubert, F.; Tosh, D.; Birnbaum, M.J.; Pende, M. Constitutively active Akt1 expression in mouse pancreas requires S6 kinase 1 for insulinoma formation. *J. Clin. Investig.* **2008**, *118*, 3629–3638, doi:10.1172/JCI35237.
19. Pelengaris, S.; Khan, M.; I Evan, G. Suppression of Myc-Induced Apoptosis in β Cells Exposes Multiple Oncogenic Properties of Myc and Triggers Carcinogenic Progression. *Cell* **2002**, *109*, 321–334, doi:10.1016/s0092-8674(02)00738-9.
20. Brubaker, P.L.; Lee, Y.C.; Drucker, D.J. Alterations in proglucagon processing and inhibition of proglucagon gene expression in transgenic mice which contain a chimeric proglucagon-SV40 T antigen gene. *J. Biol. Chem.* **1992**, *267*, 20728–33.
21. Rindi, G.; Efrat, S.; Ghatei, M.A.; Bloom, S.R.; Solcia, E.; Polak, J.M. Glucagonomas of transgenic mice express a wide range of general neuroendocrine markers and bioactive peptides. *Virchows Arch.* **1991**, *419*, 115–129, doi:10.1007/bf01600225.
22. Lines, K.E.; Nunes, R.P.V.; Frost, M.; Yates, C.J.; Stevenson, M.; Thakker, R.V. A MEN1 pancreatic neuroendocrine tumour mouse model under temporal control. *Endocr. Connect.* **2017**, *6*, 232–242, doi:10.1530/ec-17-0040.
23. Biondi, C.A.; Gartside, M.G.; Waring, P.; Loffler, K.A.; Stark, M.S.; Magnuson, M.A.; Kay, G.F.; Hayward, N.K. Conditional Inactivation of the Men1 Gene Leads to Pancreatic and Pituitary Tumorigenesis but Does Not Affect Normal Development of These Tissues. *Mol. Cell. Biol.* **2004**, *24*, 3125–3131, doi:10.1128/mcb.24.8.3125-3131.2004.
24. Wong, C.; Tang, L.H.; Davidson, C.; Vosburgh, E.; Chen, W.; Foran, D.J.; Notterman, D.A.; Levine, A.J.; Xu, E.Y. Two well-differentiated pancreatic neuroendocrine tumor mouse models. *Cell Death Differ* **2019**, *27*, 269–283, doi:10.1038/s41418-019-0355-0.
25. Glenn, S.T.; Jones, C.A.; Sexton, S.; LeVea, C.M.; Caraker, S.M.; Hajduczok, G.; Gross, K.W. Conditional deletion of p53 and Rb in the renin-expressing compartment of the pancreas leads to a highly penetrant metastatic pancreatic neuroendocrine carcinoma. *Oncogene* **2013**, *33*, 5706–5715, doi:10.1038/onc.2013.514.
26. Takano, Y.; Kasai, K.; Takagishi, Y.; Kikumori, T.; Imai, T.; Murata, Y.; Hayashi, Y. Pancreatic Neuroendocrine Tumors in Mice Deficient in Proglucagon-Derived Peptides. *PLoS ONE* **2015**, *10*, e0133812, doi:10.1371/journal.pone.0133812.
27. Saloustros, E.; Salpea, P.; Starost, M.; Liu, S.; Faucz, F.R.; London, E.; Szarek, E.; Song, W.-J.; Hussain, M.; Stratakis, C.A. Prkar1a gene knockout in the pancreas leads to neuroendocrine tumorigenesis. *Endocr. Relat. Cancer* **2017**, *24*, 31–40, doi:10.1530/erc-16-0443.
28. E Hunter, K.; Palermo, C.; Kester, J.C.; Simpson, K.; Li, J.-P.; Tang, L.H.; Klimstra, D.S.; Vlodavsky, I.; Joyce, J.A. Heparanase promotes lymphangiogenesis and tumor invasion in pancreatic neuroendocrine tumors. *Oncogene* **2013**, *33*, 1799–1808, doi:10.1038/onc.2013.142.
29. Herrera-Martinez, A.; Van, D.; Dogan, F.; Van, K.P.; Castanño, J.; Feelders, R.; Hofland, L. Utility of a 3D spheroid cell culture system in neuroendocrine tumors. *Endocr. Abstr.* **2018**, doi:10.1530/endoabs.56.p735.
30. Bresciani, G.; Hofland, L.J.; Dogan, F.; Giamas, G.; Gagliano, T.; Zatelli, M.C. Evaluation of Spheroid 3D Culture Methods to Study a Pancreatic Neuroendocrine Neoplasm Cell Line. *Front. Endocrinol.* **2019**, *10*, doi:10.3389/fendo.2019.00682.
31. Cortez, E.; Gladh, H.; Braun, S.; Bocci, M.; Cordero, E.; Björkström, N.K.; Miyazaki, H.; Michael, I.P.; Eriksson, U.; Folestad, E.; et al. Functional malignant cell heterogeneity in pancreatic neuroendocrine tumors revealed by targeting of PDGF-DD. *Proc. Natl. Acad. Sci.* **2016**, *113*, E864–E873, doi:10.1073/pnas.1509384113.
32. April-Monn, S.L.; Wiedmer, T.; Skowronska, M.S.; Maire, R.S.; Lena, M.S.; Trippel, M.; Di Domenico, A.; Muffatti, F.; Andreasi, V.; Capurso, G.; et al. 3D Primary Cell Culture: A Novel Preclinical Model For Pancreatic Neuroendocrine Tumors (PanNETs). *Neuroendocrinology* **2020**, doi:10.1159/000507669.

© 2020 by the authors. Submitted for possible open access publication under the terms and conditions of the Creative Commons Attribution (CC BY) license (http://creativecommons.org/licenses/by/4.0/).
